# Molecular Investigation of CO_2_/CH_4_ Competitive Adsorption and Confinement in Realistic Shale Kerogen

**DOI:** 10.3390/nano9121646

**Published:** 2019-11-20

**Authors:** Wenning Zhou, Zhe Zhang, Haobo Wang, Xu Yang

**Affiliations:** 1 School of Energy and Environmental Engineering, University of Science and Technology Beijing, Beijing 100083, China; Zhangzhe_111@foxmail.com (Z.Z.); whbustbseee@126.com (H.W.); xuyang@xs.ustb.edu.cn (X.Y.); 2 Beijing Key Laboratory of Energy Saving and Emission Reduction for Metallurgical Industry, University of Science and Technology Beijing, Beijing 100083, China

**Keywords:** enhanced shale gas recovery, CO_2_ sequestration, competitive adsorption, adsorption mechanism, molecular simulation

## Abstract

The adsorption behavior and the mechanism of a CO_2_/CH_4_ mixture in shale organic matter play significant roles to predict the carbon dioxide sequestration with enhanced gas recovery (CS-EGR) in shale reservoirs. In the present work, the adsorption performance and the mechanism of a CO_2_/CH_4_ binary mixture in realistic shale kerogen were explored by employing grand canonical Monte Carlo (GCMC) and molecular dynamics (MD) simulations. Specifically, the effects of shale organic type and maturity, temperature, pressure, and moisture content on pure CH_4_ and the competitive adsorption performance of a CO_2_/CH_4_ mixture were investigated. It was found that pressure and temperature have a significant influence on both the adsorption capacity and the selectivity of CO_2_/CH_4_. The simulated results also show that the adsorption capacities of CO_2_/CH_4_ increase with the maturity level of kerogen. Type II-D kerogen exhibits an obvious superiority in the adsorption capacity of CH_4_ and CO_2_ compared with other type II kerogen. In addition, the adsorption capacities of CO_2_ and CH_4_ are significantly suppressed in moist kerogen due to the strong adsorption strength of H_2_O molecules on the kerogen surface. Furthermore, to characterize realistic kerogen pore structure, a slit-like kerogen nanopore was constructed. It was observed that the kerogen nanopore plays an important role in determining the potential of CO_2_ subsurface sequestration in shale reservoirs. With the increase in nanopore size, a transition of the dominated gas adsorption mechanism from micropore filling to monolayer adsorption on the surface due to confinement effects was found. The results obtained in this study could be helpful to estimate original gas-in-place and evaluate carbon dioxide sequestration capacity in a shale matrix.

## 1. Introduction

As an alternative to conventional natural gas resources, shale gas receives considerable attention due to its vast resource base and wide distribution around the world. However, the exploitation and development of shale gas is a major challenge because of its complex structure and the ultra-low permeability of shale reservoirs [1,2]. In addition to typical hydraulic fracturing [3], supercritical carbon dioxide (SC-CO_2_) was recently proposed as an alternative fracturing fluid to achieve CO_2_ sequestration simultaneously with enhanced shale gas recovery (CS-EGR) in shale gas reservoirs [4]. This technology is considered as one of the most promising techniques of carbon capture, utilization, and storage (CCUS) [5].

Shale gas in reservoirs is mainly composed of free gas in intergranular pores and natural fractures, adsorbed gas in an organic matrix, and clay minerals, as well as dissolved gas in liquid. The adsorbed gas could take up 20–80% of the total gas-in-place (GIP). This percentage could be as high as 60–85% for organic-rich shale [6]. Previous studies suggested that the total GIP in shale reservoirs is affected by the total organic carbon (TOC) content, organic matter type, thermal maturity, and pore structure [7,8]. Wu et al. carried out adsorption experiments of light hydrocarbons and carbon dioxide on shale samples and isolated kerogen, respectively [9]. They found that the contribution of kerogen on the adsorption capacity could account for over 50 wt.%, although the TOC takes only 3.65 wt.% of shale samples. Meanwhile, they also pointed out that the contribution of inorganic matter for adsorption capacity cannot be ignored due to its large surface area. Ross and Bustin examined a few shale samples with different thermal maturation and reported positive correlations between CH_4_ adsorption capacity and TOC; however, the ratios were different for different shales [10]. However, Gasparik et al. [11] and Li et al. [12] later found that the positive correlation between adsorption capacity and TOC would reverse at very high maturities of shale formations. Zhou et al. experimentally measured the adsorption capacities of CO_2_ and CH_4_ on a set of shale samples which were collected from the Sichuan and Ordos Basins, China [13]. They claimed that the preferential adsorption ratio of carbon dioxide over methane varies between 1.66 and 8.32 in shale formations. In addition to organic type and thermal maturity, pore structures of the shale matrix are also key factors for gas adsorption. To better understand the nature of pore structures of shale formations, researchers applied many techniques, such as small-angle neutron scattering (SANS), field-emission scanning electron microscopy (FE-SEM), microcomputed tomography (μ-CT), mercury intrusion porosimetry (MIP), and low-pressure N_2_/CO_2_ adsorption experiments, to obtain the pore size distribution (PSD) of shale reservoirs [14,15,16,17,18]. It was found that shale formations have multiscale pore size distribution with numerous micropores (<2 nm), mesopores (2–50 nm), and macropores (>50 nm). Among them, micropores and fine mesopores (<10 nm) are the major contributors to the total surface area which is favorable for gas adsorption in shale reservoirs [19]. Therefore, the study of gas adsorption mechanism and competitive adsorption behavior of a CO_2_/CH_4_ mixture in kerogen nanopores is of significant importance for CS-EGR projects in shale gas reservoirs.

In addition to experimental studies, molecular simulation is considered as an effective tool to gain microscopic insights into complex physical phenomena or processes in many research areas [20,21,22,23,24]. The adsorption behaviors in nanopores of shale reservoirs were investigated via molecular simulation by many researchers [25,26,27,28]. The effects of temperature, pressure, and pore size on the adsorption performance of pure methane and a CO_2_/CH_4_ mixture in the slit pore of shale formations were examined. Lin et al. used graphene to simplify the organic matter of a shale matrix and found that the adsorption isotherms of CH_4_ in a graphene slit follow a monolayer Langmuir equation [29]. Also, by using graphene slit model, Chen et al. [30] and Zhang et al. [31] studied the effect of pore size on the adsorption performance of methane. They found that there is a threshold of slit pore size under which 100% of CH_4_ is in an adsorbed state without free gas in the confined nanopores. The effects of pressure, temperature, and moisture content on the competitive adsorption behavior of a CO_2_/CH_4_ mixture were also examined in graphene slit nanopore [32,33,34,35]. Their results revealed that CO_2_ molecules are preferentially adsorbed over CH_4_ onto the surface of graphene nanopore. In addition to the graphene slit model, other carbon-based models, such as carbon nanotubes, triangular pores, and square pores were constructed to represent different shapes of nanopores in shale organic matter [26,36,37,38]. In order to take account the into maturation level of organic matter, different types of functional groups were constructed on the graphene surface [39,40]. Their results indicated that the functional group has an obvious impact on the adsorption capacity and selectivity of CO_2_ over CH_4_. Additionally, the adsorption behavior and confinement effects of pure CH_4_ and a CO_2_/CH_4_ mixture were examined in inorganic nanopores [41,42,43]. The effects of pressure and pore size on nanoconfinement and the gas adsorption mechanism were explored and discussed in their work.

As a matter of fact, kerogen is a highly complex and heterogeneous organic matter in shale reservoirs [44]. To take into consideration the complicated chemical characteristics of kerogen, a number of realistic kerogen molecular models were proposed based on experimental data [45,46,47,48]. Wang et al. applied the grand canonical Monte Carlo method (GCMC) to examine the effects of pressure, temperature, and mole fraction on the adsorption isotherms and adsorption selectivity of a CO_2_/CH_4_ binary mixture in type I-A kerogen [49]. They claimed that the adsorption capacity of CO_2_ in shale kerogen is stronger than that of CH_4_ and obtained the optimal injection depth of 1000–2500 m for supercritical carbon dioxide-enhanced shale gas exploitation in shale reservoirs. Pathak et al. found that the CO_2_ is more strongly retained than CH_4_ in the bulk type II kerogen matrix, which is favorable for the sequestration of carbon dioxide in shale formations [50]. Huang et al. studied the effects of organic type and water content on the competitive adsorption behaviors of a CH_4_ and CO_2_ mixture [51]. They reported that water content has a negative effect on the gas adsorption capacity and that kerogen III-A is the optimal organic type for a CS-EGR project. By inserting an empty space into two kerogen matrices to construct a realistic kerogen slit, the characteristics of the chemical components and nanopore shape can be captured [52]. However, they assumed that gas adsorption in the slit kerogen model is based on monolayer adsorption. Although much work was carried out, the microscopic adsorption mechanism of a CO_2_/CH_4_ mixture and the confinement effects in realistic kerogen nanopores remain unclear.

In this work, we extended our previous studies on CO_2_/CH_4_ competitive adsorption in graphene and kaolinite clay slit nanopores of shale reservoirs [32,41]. Firstly, different kerogen molecular models developed by Ungerer et al. [45] were employed to construct a bulk kerogen matrix with different maturity levels. By applying molecular dynamics and the grand canonical Monte Carlo method, the effects of temperature, pressure, and maturity on the adsorption behavior pure CH_4_ and CO_2_/CH_4_ mixture were explored. Furthermore, to characterize the nanopore structure of organic matter of a shale matrix, a realistic slit kerogen nanopore was developed. The effects of pore size on the adsorption behavior and confinement effects were examined and discussed.

## 2. Materials and Methodology

### 2.1. Construction of Kerogen Models

**Figure 1:**
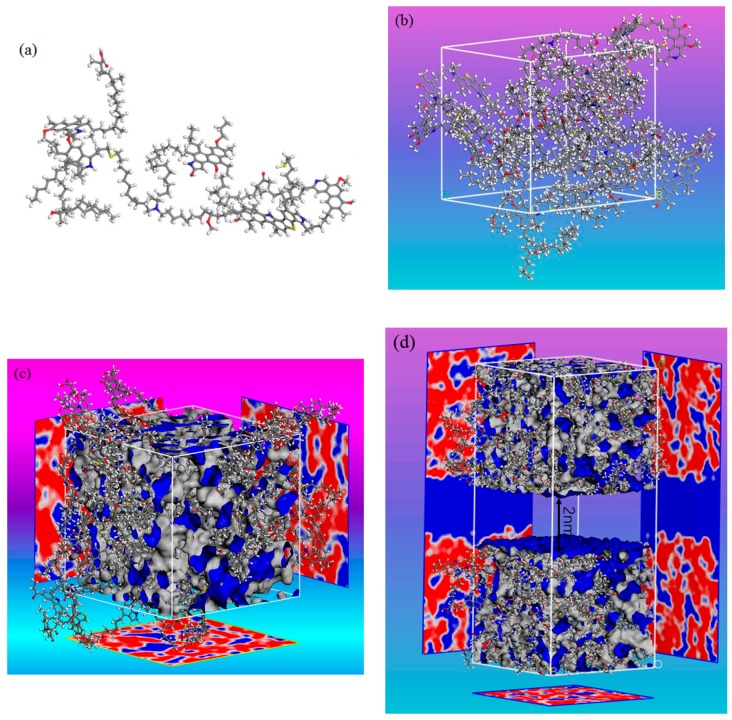
(**a**) Molecular model of type I-A kerogen molecule with a chemical formula C_251_H_385_O_13_N_7_S_3_; (**b**) bulk kerogen configuration with 10 kerogen molecules; (**c**) bulk kerogen model with porosity; (**d**) structure of a realistic slit kerogen nanopore.

Kerogen formations can be mainly classified as types I, II, and III based on their carbon (C), hydrogen (H), and oxygen (O) contents [45]. In this study, five kerogen molecular models, i.e., I-A, II-A, II-B, II-C, and II-D, proposed by Ungerer et al. [45], representing different maturity levels were adopted. Type I-A kerogen is derived from immature Green River shale, which is usually correlated with oil shale retorting and shale oil. Type II kerogen is obtained from the Duvernay series, which is the common source of shale gas. These kerogen samples show an increasing maturity from II-A (immature) to II-D (postmature). The molecular configuration of the type I-A kerogen model is shown in Figure 1a. To build bulk kerogen models, 10 kerogen units were placed in a cubic simulation box with periodic boundary conditions, as seen in Figure 1b. The geometry optimization and annealing dynamics were carried out to relax kerogen models. The annealing dynamics simulations were carried out using the canonical ensemble (constant atom number, volume and temperature, NVT). A total of 10 annealing cycles were adopted with temperature increasing from 300 to 800 K, and a total simulation time of 400 ps was executed to obtain stable structures with the lowest energy. Figure 1c shows the kerogen model with porosity, which was obtained by helium probe. The densities and porosities of the proposed kerogen model varied from 1.01–1.18 g/cm^3^ and 10.8–19.6%, which are consistent with previous experimental and simulation work [51]. In order to characterize the pore nature of realistic kerogen, a slit kerogen model was built by inserting an empty space into two kerogen matrices [53], as shown in Figure 1d. The Accelrys Materials Studio software was applied for kerogen model construction and all simulation cases in this study.

### 2.2. GCMC Simulation Details

In the present work, the GCMC method was employed to examine the gas adsorption behavior and mechanism in kerogen of shale formations. The COMPASS force field was adopted to perform all the simulations in this study [54]. The Monte Carlo steps involved in the GCMC simulations are random creations, destructions, rotations, and translations. These trial moves are accepted or rejected by the Metropolis acceptance criterion. Nonbonding interactions are represented by the van der Waals (vdW) potential and electrostatic potential. The vdW interactions described by a Lennard–Jones (LJ) 9-6 potential were determined using the atom-based method with a fine cutoff distance of 15.5 Å, while the electrostatic interactions were obtained by the Ewald method with an accuracy of 10^−3^ kcal/mol, which can be described by Equation (1).

(1) $$u\left(r_{ij}\right)=u^{LJ}+u^{C}=\varepsilon_{ij}\left[2{\left(\frac{\sigma_{ij}}{r_{ij}}\right)}^{9}-3{\left(\frac{\sigma_{ij}}{r_{ij}}\right)}^{6}\right]+\frac{q_{i}q_{j}}{4\pi \varepsilon_{0}r_{ij}},$$

where *r_ij_* denotes the distance between atom *i* and *j*, *ε*_0_ is the relative dielectric constant, *q_i_* and *q_j_* represent the charges of atom *i* and *j*, and *ε_ij_* and *σ_ij_* are the LJ well depth and LJ size, respectively, which can be obtained by a sixth power rule [55].

(2) $$\varepsilon_{ij}={\left(\varepsilon_{0,ii}\varepsilon_{0,jj}\right)}^{1/2}\left[2\sigma_{0,ii}{}^{3}\sigma_{0,jj}{}^{3}/(\sigma_{0,ii}{}^{6}+\sigma_{0,jj}{}^{6})\right],$$

(3) $$\sigma_{ij}={\left((\sigma_{0,ii}{}^{6}+\sigma_{0,jj}{}^{6})/2\right)}^{1/6},$$

A total of 1 × 10^7^ Monte Carlo steps were carried out for each simulation case. The first 5 × 10^6^ steps were performed to relax the system. The last 5 × 10^6^ steps were utilized to calculate the required thermodynamic variables. In GCMC simulations, chemical potential, volume, and temperature are independent parameters. Moreover, the chemical potential is calculated as a function of fugacity instead of pressure. Within the current work, the fugacity (i.e., “corrected” pressure) was determined by employing the Peng–Robinson equation of state (PR EOS) [56]. To compare the simulation results with experimental data, the simulated absolute adsorption capacity can be converted to excess adsorption capacity using the following expression:

(4) $$n^{\mathrm{ex}}=n^{\mathrm{abs}}-\rho^{b}V_{\mathrm{ads}},$$

where n^ex^ and n^abs^ denote the excess and absolute adsorption amounts, respectively. *ρ*^b^ is the density of the bulk phase, which can be calculated using the PR equation of state. *V*_ads_ represents the pore volume.

In order to investigate and quantify the competitive adsorption behavior of a CO_2_/CH_4_ mixture, the adsorption selectivity of CO_2_ over CH_4_ is introduced.

(5) $$S_{{\mathrm{CO}}_{2}/{\mathrm{CH}}_{4}}=\frac{x_{{\mathrm{CO}}_{2}}/x_{{\mathrm{CH}}_{4}}}{y_{{\mathrm{CO}}_{2}}/y_{{\mathrm{CH}}_{4}}},$$

where *x_i_* and *y_i_* denote the average mole fraction of component *i* in the adsorption phase and bulk phase, respectively. It indicates the preferential adsorption of CO_2_ over CH_4_ in kerogen pores when $S_{{\mathrm{CO}}_{2}/{\mathrm{CH}}_{4}}>1$. For more detailed information regarding GCMC simulation, one can refer to our previous work [23,32,41].

## 3. Results and Discussion

### 3.1. Model Validation

**Figure 2:**
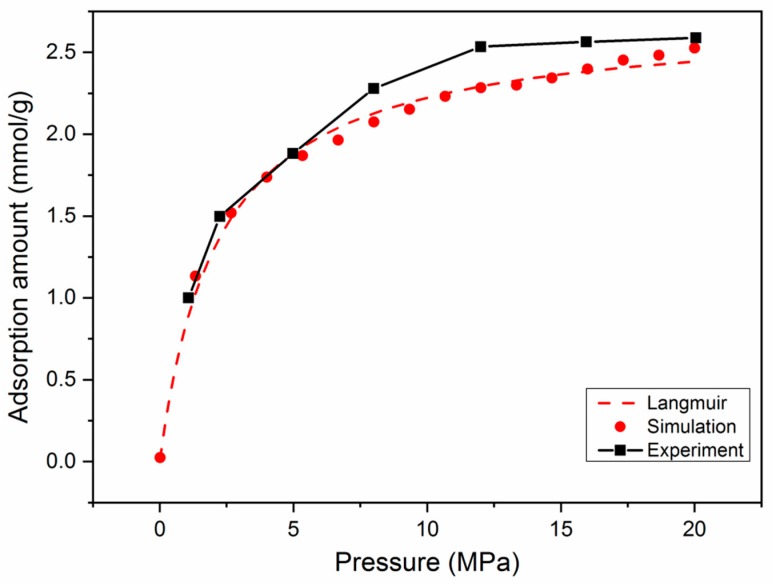
Comparison between simulation results, experimental data, and theoretical Langmuir fitting.

Firstly, to ensure the validity of the model, the simulation results of a pure CH_4_ absolute adsorption isotherm in type II-A kerogen were compared with experimental data [39], as displayed in Figure 2. It can be observed that the comparison shows satisfactory agreement. Furthermore, the simulation results were also found to fit very well with the Langmuir equation, which is widely applied to describe gas adsorption behavior in shale formations [32,52]. It should be noted that, in addition to temperature and pressure conditions, the adsorption behavior of CH_4_ in kerogen is also affected by the maturity level and moisture content. The adsorption isotherms would differ in kerogen with different maturity levels. Nevertheless, the agreement between the simulated and experimental results verified the model in this study.

### 3.2. Effect of Temperature and Pressure on Adsorption Behavior

**Figure 3:**
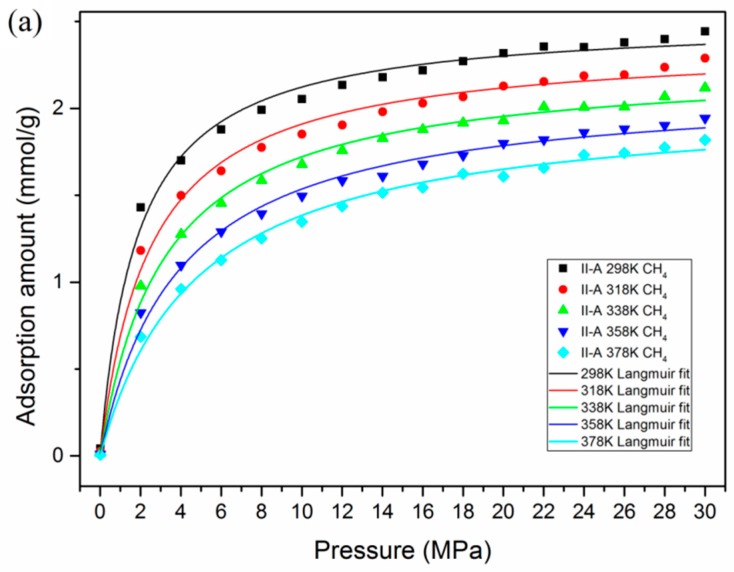

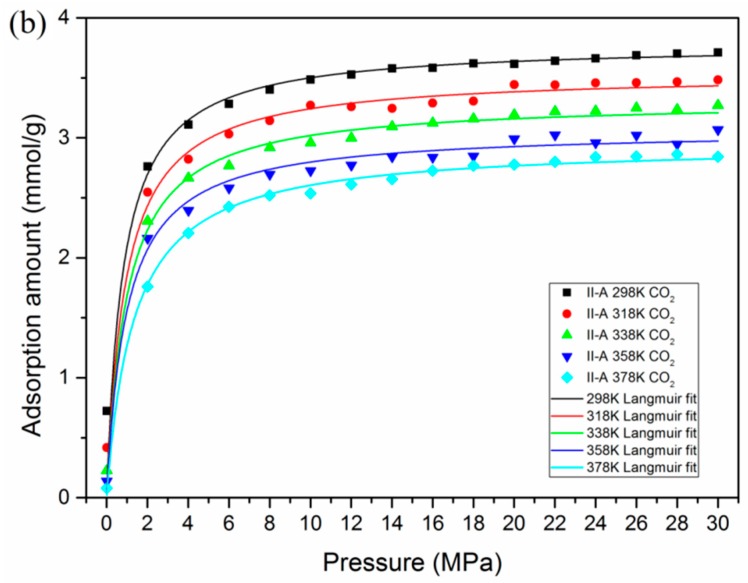
The adsorption isotherms of CH_4_ and CO_2_ at different temperatures and Langmuir fitting: (**a**) CH_4_; (**b**) CO_2_.

In this section, a series of simulation cases were carried out to study the effects of temperature and pressure on the gas adsorption behavior in type II-A kerogen. Figure 3 presents the adsorption isotherms for pure CH_4_ and pure CO_2_ at different temperatures. It was found that the adsorption of CH_4_ and CO_2_ in shale organic matter exhibits Langmuir type I monolayer adsorption. For both CH_4_ and CO_2_, the adsorption capacities decreased with the increase in temperature. With increasing pressure, the adsorption amounts increased, with CO_2_ reaching its maximum capacity more quickly than CH_4_. Under the same conditions of temperature and pressure, the adsorption amount of CO_2_ was much larger than that of CH_4_. These findings are in line with previous studies [51,57]. Additional results regarding the adsorption isotherms of CH_4_ in kerogen with different maturity level (i.e., I-A, II-B, II-C, and II-D) can be found in Figure S1 (Supplementary Materials). A similar trend of adsorption isotherm was found in kerogen with different maturity levels, but the adsorption capacities varied.

**Figure 4:**
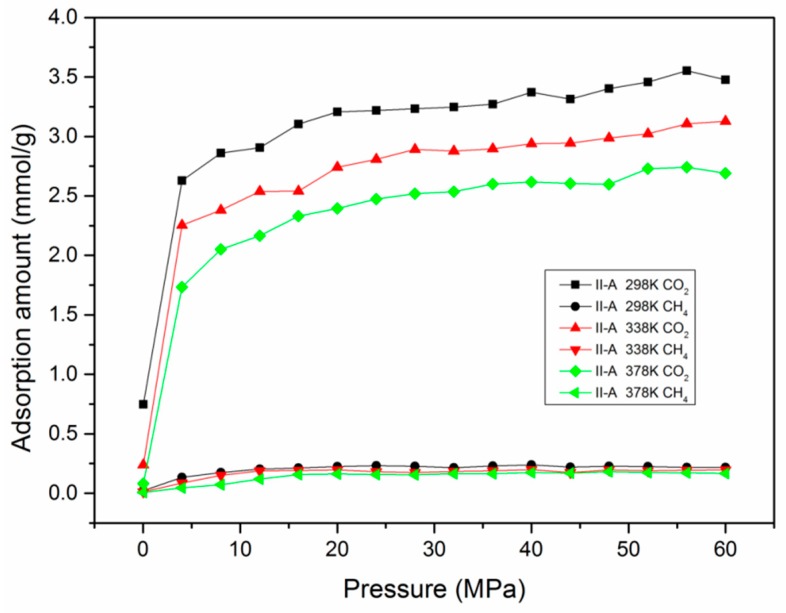
The adsorption amounts of a CO_2_/CH_4_ binary mixture in type II-A kerogen with pressure at different temperatures.

To further study the effect of temperature and pressure on competitive adsorption behavior, a series of CO_2_/CH_4_ mixture adsorption simulation cases were carried out. Figure 4 presents the adsorption isotherms of a CO_2_/CH_4_ binary mixture under different temperatures in a type II-A kerogen matrix. The simulation results demonstrate that the adsorption capacity of CH_4_ was greatly suppressed in the presence of CO_2_. This can be ascribed to the intrinsic quadrupole moment of CO_2_ which could result in strong interactions between CO_2_ molecules and the surface of shale organic matter [32,58]. For example, the adsorption capacity of CO_2_ was 2.538 mmol/g under the conditions of 12 MPa and 338 K, while it was 0.189 mmol/g for CH_4_.

**Figure 5:**
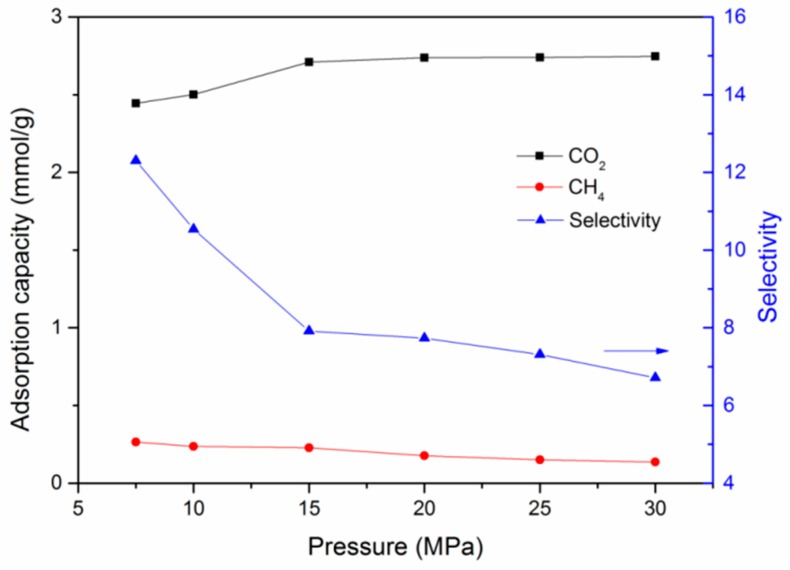
Variation of adsorption capacities and selectivity of a CO_2_/CH_4_ binary mixture as a function of CO_2_ partial pressure (CH_4_ partial pressure fixed at 10 MPa, T = 298 K).

As known, the injection of carbon dioxide also plays an essential role in the performance of shale gas development and carbon dioxide sequestration. To quantify this influence, a series of cases with different CO_2_ injection pressure were performed for a fixed partial pressure of CH_4_. The simulation results indicate that with the increase of CO_2_ partial pressure, the adsorption amount of CO_2_ increased significantly at first and then gradually after a certain pressure, as displayed in Figure 5. CH_4_ had a gradual reduction with the increasing injection pressure of CO_2_. The selectivity of CO_2_/CH_4_ showed a significant and then gradual reduction with the increase in CO_2_ injection pressure. This means that an increase in CO_2_ injection pressure would be helpful for the storage of CO_2_ in practical CS-EGR projects in shale gas reservoirs, but there exists an optimal value above which the storage efficiency would not increase much. However, a much lower pressure could be favorable for the enhancement of selectivity of CO_2_/CH_4_, which is consistent with the results of previous studies [32,40,59]. Additional results regarding different CH_4_ partial pressures (i.e., 5 MPa, 15 MPa, 20 MPa, and 25 MPa) can be found in Figure S2 (Supplementary Materials).

### 3.3. Effect of Maturity on Adsorption Behavior

**Figure 6:**
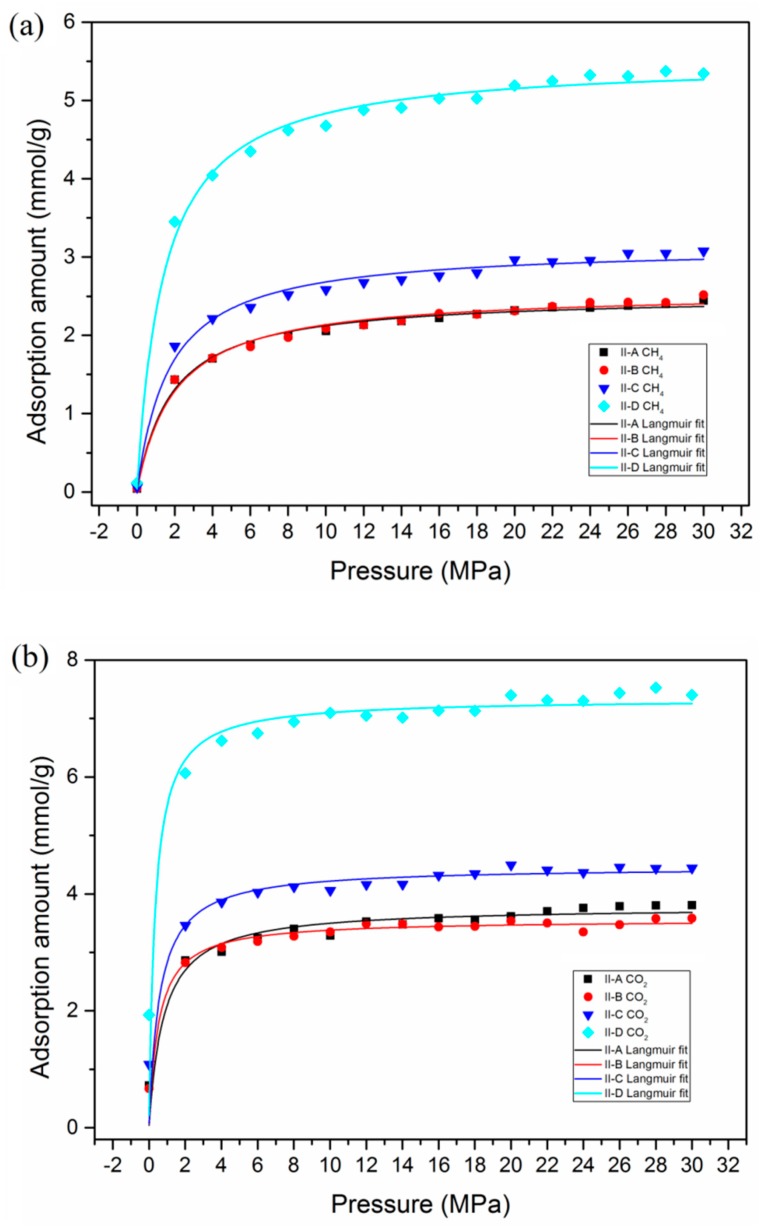
The adsorption isotherms of CH_4_ and CO_2_ in kerogen with different maturity levels and Langmuir fitting: (**a**) CH_4_; (**b**) CO_2_.

In order to investigate the effects of type and maturity level on the adsorption behavior, the adsorption isotherm simulations of CH_4_ and CO_2_ in kerogen with different maturity levels (II-A, II-B, II-C, and II-D) were carried out. It can be observed that, with increasing maturity, the adsorption capacities of both CH_4_ and CO_2_ tended to increase, as presented in Figure 6. This is due to the fact that the kerogen matrices with different maturity have different C/O and C/H ratios, which has a great impact on the gas adsorption behavior in kerogen. It is found that types II-A and II-B kerogen had similar adsorption capacities. However, type II-D exhibited an obvious superiority in the adsorption capacities of CH_4_ and CO_2_. Similarly, organic-rich shale reservoirs with high maturity level were reported as the optimized organic type for CS-EGR projects [51].

### 3.4. Effect of Moisture Content on Adsorption Behavior

**Figure 7:**
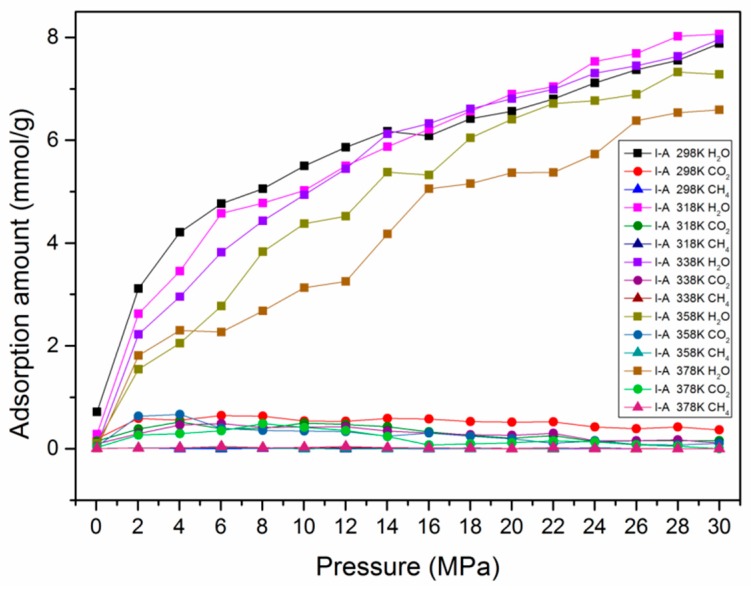
The adsorption amounts of H_2_O, CH_4_, and CO_2_ with pressure in type I-A kerogen at different temperatures.

**Figure 8:**
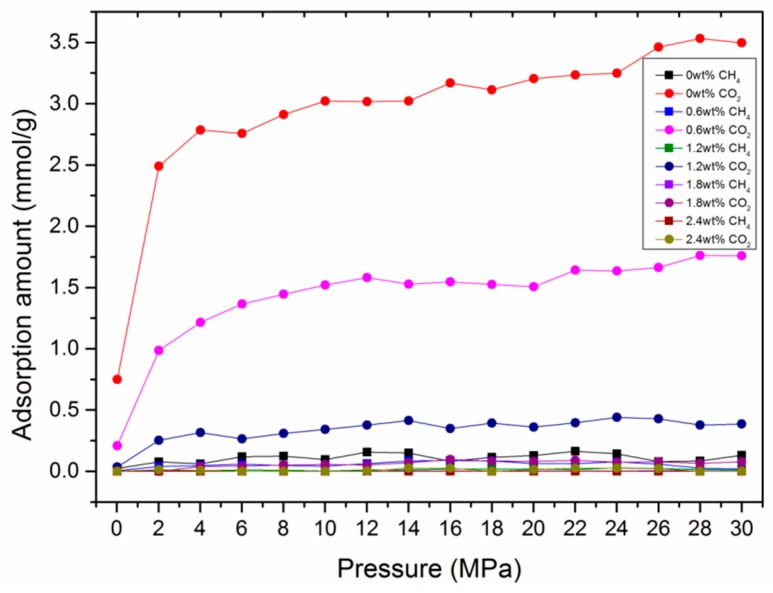
The adsorption amounts of CH_4_ and CO_2_ with pressure in type I-A kerogen at different moisture contents.

In actual shale reservoirs, moisture content usually exists in organic matter, which was reported to have great influence on gas adsorption performance in previous work [59,60,61,62]. To qualify the effect of moisture content on the adsorption behavior in kerogen, a certain amount of H_2_O molecules, representing 0−2.4 wt.% moisture content, were preloaded in the developed kerogen models. The results show that the adsorption ability of H_2_O on the kerogen surface was much stronger than that of CO_2_ and CH_4_ at various temperatures, as can be seen in Figure 7. This can be attributed to the stronger polarity of H_2_O molecules over CO_2_ and CH_4_ molecules, causing H_2_O molecules to occupy adsorption sites more easily. The quantities of suppression can be found in Figure 8. With the presence of H_2_O molecules, the adsorption capacities of CO_2_ and CH_4_ were significantly suppressed. It can be observed that the adsorption capacities of CO_2_ and CH_4_ dropped from 1.547 mmol/g and 0.089 mmol/g in dry kerogen to 0.096 mmol/g and 0.001 mmol/g in a kerogen matrix with a moisture content of 1.8 wt.%, respectively.

### 3.5. Adsorption Behavior and Confinement in Realistic Kerogen Nanopore

**Figure 9:**
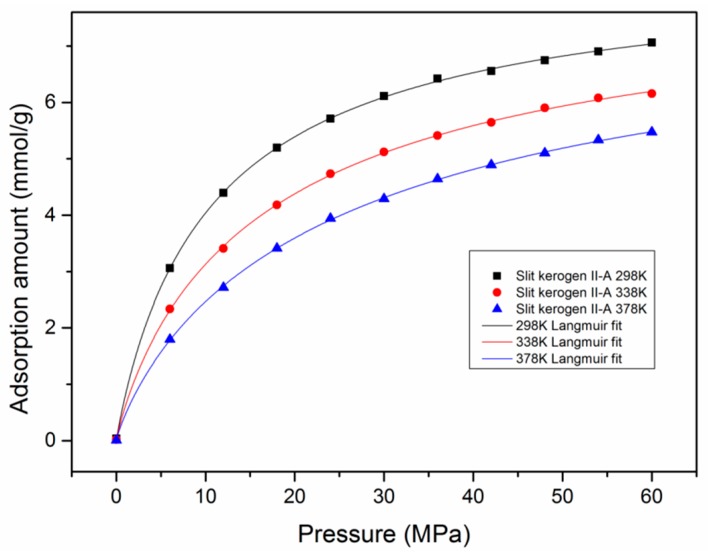
The adsorption isotherms of pure CH_4_ and Langmuir fitting in a type II-A slit kerogen nanopore with a pore size of 2 nm at different temperatures.

In order to explore the adsorption behavior and confinement effects in realistic kerogen, a few simulation cases were conducted in the developed slit kerogen nanopore. Figure 9 shows the adsorption isotherms of pure CH_4_ in type II-A slit kerogen nanopore with a pore size of 2 nm at different temperatures. It can be observed that the adsorption isotherms were similar to that of bulk kerogen. With the increment of temperature, the adsorption capacity decreased. In addition, the results show that the adsorption isotherms also fit the Langmuir equation well.

**Figure 10:**
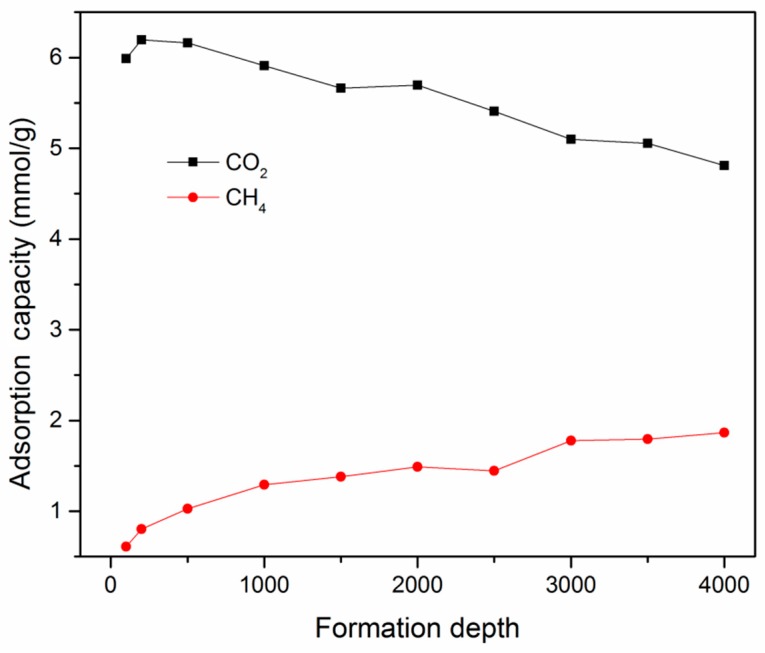
Variation of adsorption capacities of a CO_2_/CH_4_ binary mixture as a function of buried depth of shale formation (mole fraction of CH_4_ = 0.5).

Figure 10 presents the competitive adsorption behavior of a CO_2_/CH_4_ binary mixture as a function of buried depth of shale formation. The geological pressure and temperature as a function of depth can be determined by the pressure gradient of 15 MPa/km and the geothermal gradient of 27.3 °C/km [63]. It can be seen from the figure that, with increasing formation depth, the adsorption capacity of CO_2_ increased at first to reach its maximum capacity and then gradually decreased. On the other hand, for CH_4_, the adsorption capacity tended to increase gradually. This is due to the fact that both pressure and temperature have an impact on gas adsorption capacity in shale reservoirs. As discussed before, the adsorption capacity increased with the increment in pressure, while it decreased with the increasing temperature. In a shale reservoir at shallow buried depth, the pressure had a dominant role in determining the adsorption capacity of CO_2_. With increasing buried depth, the effect of temperature tended to be significant, causing the decrease of adsorption capacity of CO_2_. On the other hand, for CH_4_, the effect of pressure was dominant in the competitive situation with CO_2_. These findings might be helpful in designing and optimizing CS-EGR projects in shale gas reservoirs. It should be noted that the studied kerogen matrix was considered rigid in this study. The kerogen swelling during the adsorption process also has an impact on gas adsorption and transport behaviors [64], which cannot be ignored in actual CS-EGR projects.

**Figure 11:**
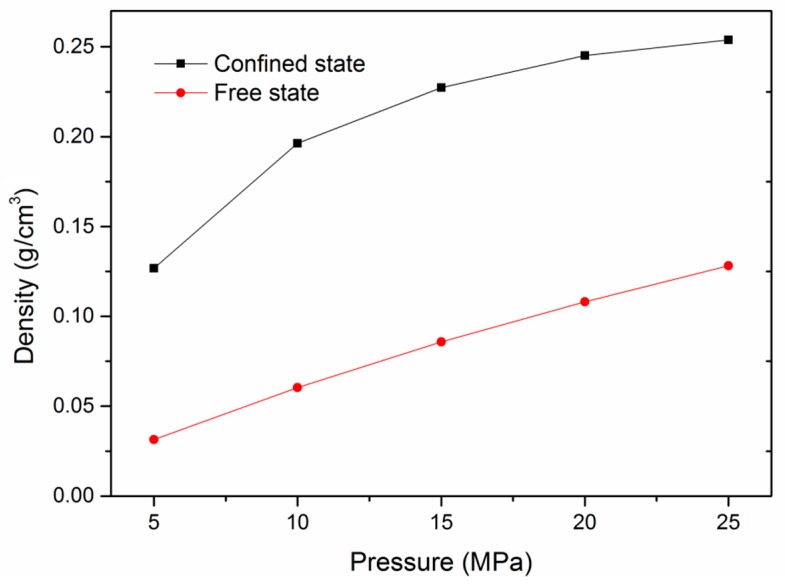
Comparison of density between the confined state and the free state of pure CH_4_ as a function of pressure in a slit kerogen nanopore with a pore size of 2 nm (T = 298 K).

The knowledge of adsorption mechanism in kerogen nanopores is important in predicting gas adsorption in shale reservoirs; however, few studies focused on this. To explore the adsorption mechanism in kerogen nanopores, a few more simulation cases were carried out in slit kerogen nanopores. Figure 11 plots the comparison of density between the adsorbed state under confinement and the free state of CH_4_ in slit kerogen nanopore with a pore size of 2 nm. It can be observed that, in the pressure range of 5–25 MPa, which is higher than the critical pressure of CH_4_ (4.64 MPa), the densities of adsorbed gas were much larger than that in the free state. This means that CH_4_ molecules under supercritical pressure were in an adsorbed state in the slit kerogen nanopore with a pore size of 2 nm, indicating that micropore filling is the dominant adsorption mechanism. These results are consistent with previous work on adsorption in kaolinite and montmorillonite clay nanopore in shale reservoirs [41,42]. It should be mentioned that the densities of CH_4_ in the free state were calculated by molecular simulations under the same conditions of pressure and temperature in an unconfined box in this work.

**Figure 12:**
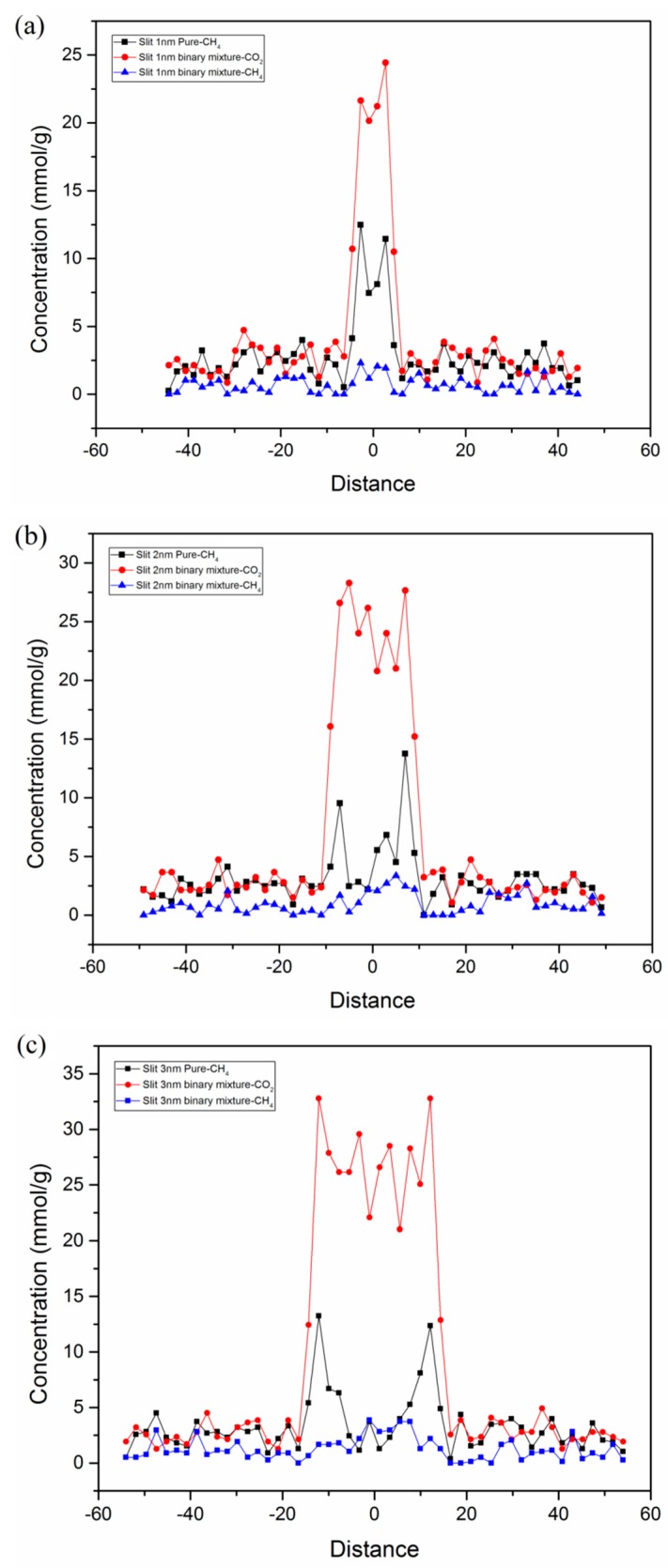
Concentration profile of pure CH_4_ and a CO_2_/CH_4_ binary mixture adsorption in slit kerogen nanopores: (**a**) 1 nm; (**b**) 2 nm; (**c**) 3 nm (partial pressures of CO_2_ and CH_4_ were 10 MPa and 5 MPa, respectively; T = 298 K).

In addition, the competitive adsorption behaviors and confinement effects of CO_2_/CH_4_ in a slit kerogen nanopore were examined. Figure 12 displays the concentration profile of pure CH_4_ and a CO_2_/CH_4_ binary mixture in terms of competitive adsorption in a slit kerogen nanopore with different pore sizes. It was found that the slit pore contributed a large amount of adsorption capacity due to its large surface area. The adsorption capacity of CH_4_ was greatly suppressed with the presence of CO_2_ in slit kerogen pores. For pure CH_4_, it can be observed that there were two obvious primary adsorption layers adjacent to the kerogen surface in 3-nm slit nanopore. The concentration decreased at the center of the nanopore. However, this situation was different for the slit kerogen nanopores of 1 nm and 2 nm without an obvious reduction in concentration at the center position on the nanopore. This indicates that confinement effects existed in slit kerogen nanopores and small mesopores, and the decline of pore size and increment of pressure could enhance the confinement. Therefore, for pure CH_4_, the adsorption mechanism in a slit kerogen nanopore (pore size < 2 nm) is mainly micropore filling due to confinement. On the other hand, for larger nanopores, the adsorption mechanism is dominated by Langmuir monolayer adsorption. The adsorption mechanisms of micropore filling and monolayer coexist in slit kerogen nanopores. It should be noted that the pore size of 2 nm obtained in this study, which determined different dominant adsorption mechanisms in kerogen nanopore, was related the other parameters, including temperature, pressure, competitive condition, etc. Nevertheless, it can be concluded that there exists a certain value of pore size, which is dependent on the chemical composition and geological conditions of shale reservoirs. Beyond this value, the dominant adsorption mechanism would transit from micropore filling to monolayer adsorption. These findings will provide theoretical support in predicting accurate shale gas adsorption capacity and CO_2_ sequestration potential according to the pore structure analysis of shale reservoirs.

## 4. Conclusions

In this work, the adsorption mechanism and competitive adsorption behavior were explored by using the grand canonical Monte Carlo method. The effects of temperature, pressure, maturity level, moisture content, and pore size on the adsorption mechanism, as well as competitive adsorption performance, were discussed in detail. Major conclusions can be summarized as follows:

(1) At various conditions, CO_2_ is preferentially adsorbed over CH_4_ in kerogen of shale formations. A lower temperature and higher pressure are favorable for the adsorption capacity of CO_2_ and CH_4_. However, a much lower pressure would be beneficial for the adsorption selectivity of CO_2_ over CH_4_.
(2) Higher maturity of organic matter in shale reservoirs exhibits superior adsorption capacities of both CO_2_ and CH_4_. The presence of water content is unfavorable for the adsorption capacities of CO_2_ and CH_4_. Compared with dry kerogen, the adsorption capacities of CO_2_ and CH_4_ dropped from 1.547 mmol/g and 0.089 mmol/g to 0.096 mmol/g and 0.001 mmol/g in kerogen with a moisture content of 1.8 wt.%, respectively.
(3) Confinement effects exist in slit kerogen micropores (<2 nm) and small mesopores (~3 nm), and the decline of pore size and increment of pressure (larger than supercritical pressure) could enhance the confinement.
(4) The adsorption mechanisms of miropore filling and monolayer adsorption coexist in slit kerogen nanopores due to confinement. For pore sizes smaller than 2 nm, the adsorption in slit kerogen nanopores is mainly caused by miropore filling. On the other hand, for larger pore sizes, the adsorption mechanism is dominated by Langmuir monolayer adsorption in slit kerogen nanopores.

## Supplementary Materials

The following are available online at https://www.mdpi.com/2079-4991/9/12/1646/s1: Figure S1: The adsorption isotherms of pure CH_4_ at different temperatures and Langmuir fitting in kerogen with different maturity levels: (a) I-A; (b) II-B; (c) II-C; (d) II-D; Figure S2: Variation of adsorption capacities and selectivity of a CO_2_/CH_4_ binary mixture as a function of CO_2_ partial pressure at different CH_4_ fixed partial pressures: (a) 5 MPa; (b) 15 MPa; (c) 20 MPa; (d) 25 MPa, T = 298 K.
