# Supplementary material for: Molecular Investigation of CO2/CH4 Competitive Adsorption and Confinement in Realistic Shale Kerogen
Source: Nanomaterials (Basel). 2019 Nov 20;9(12):1646. doi: 10.3390/nano9121646 (PMC6956192; doi:10.3390/nano9121646)
Supplement: Supplementary file 1 [file nanomaterials-09-01646-s001.pdf]

nanomaterials

MDPI

Article

Molecular Investigation of CO_2/CH_4 Competitive Adsorption and Confinement in Realistic Shale Kerogen

Wenning Zhou [1,2,*], Zhe Zhang [1], Haobo Wang [1] and Xu Yang [1]

[1] School of Energy and Environmental Engineering, University of Science and Technology Beijing, Beijing 100083, China; Zhangzhe_111@foxmail.com (Z.Z.); whbustbseee@126.com (H.W.); xuyang@xs.ustb.edu.cn (X.Y.)

[2] Beijing Key Laboratory of Energy Saving and Emission Reduction for Metallurgical Industry, University of Science and Technology Beijing, Beijing 100083, China

* Correspondence: wenningzhou@ustb.edu.cn; Tel.: +86-10-6233-2730

List of Figures

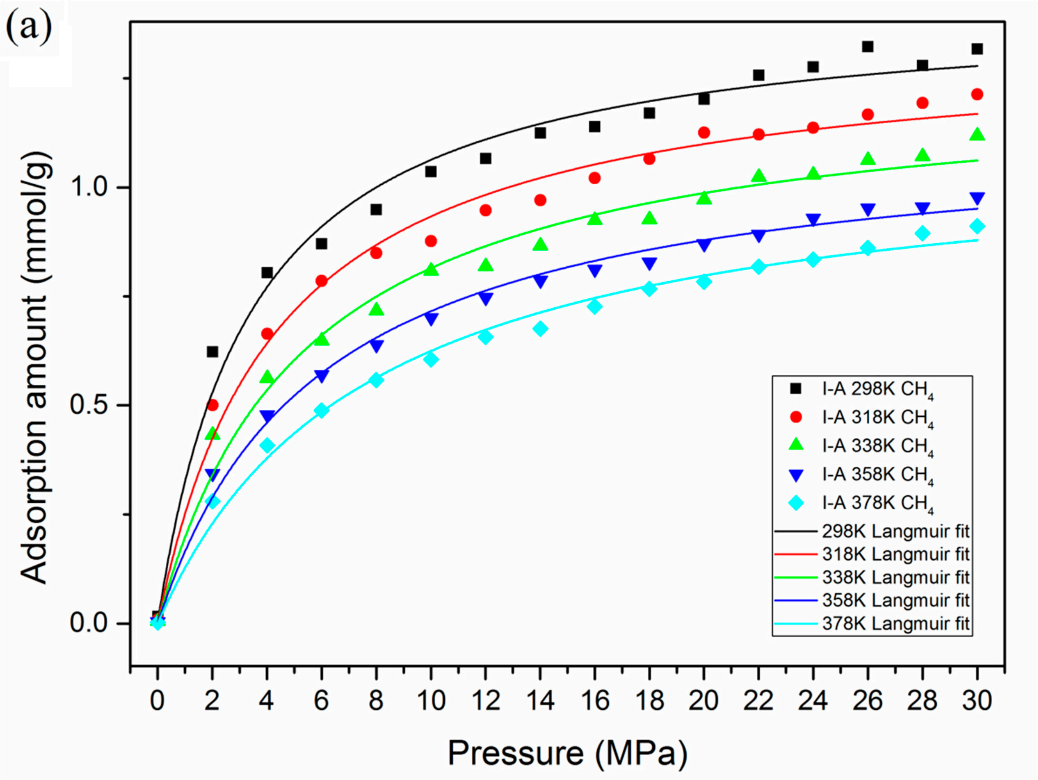

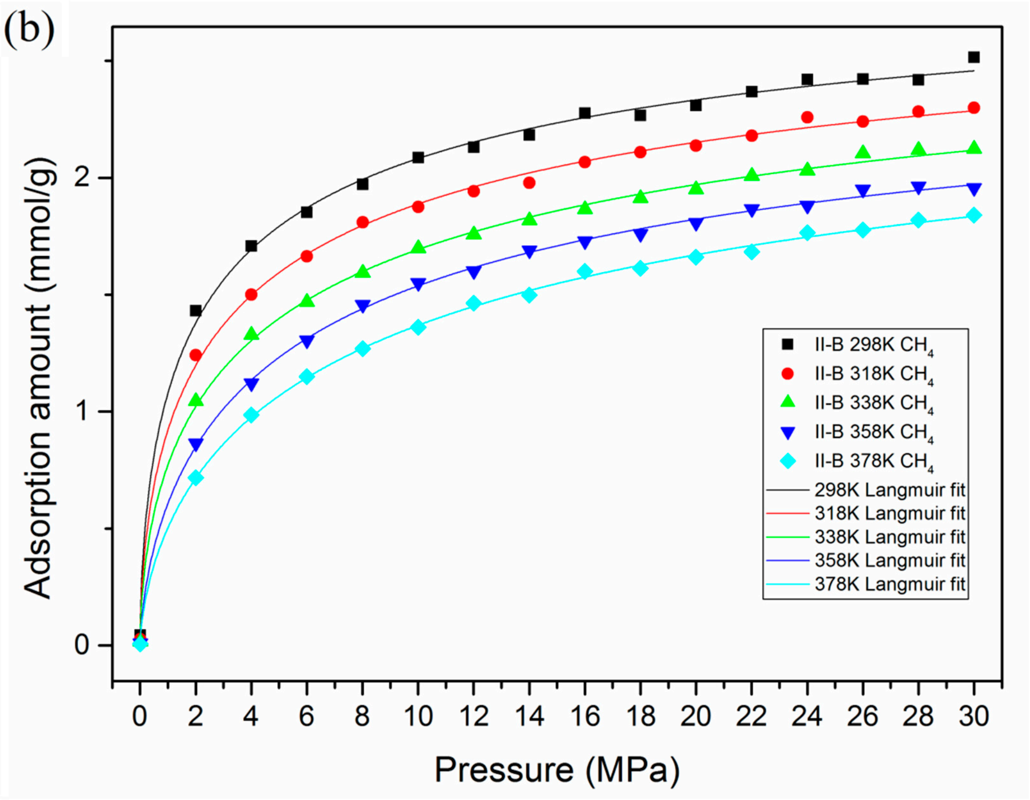
(b)
Adsorption amount (mmol/g)
Pressure (MPa)
0
1
2
0 2 4 6 8 10 12 14 16 18 20 22 24 26 28 30
II-B 298K CH4
II-B 318K CH4
II-B 338K CH4
II-B 358K CH4
II-B 378K CH4
298K Langmuir fit
318K Langmuir fit
338K Langmuir fit
358K Langmuir fit
378K Langmuir fit

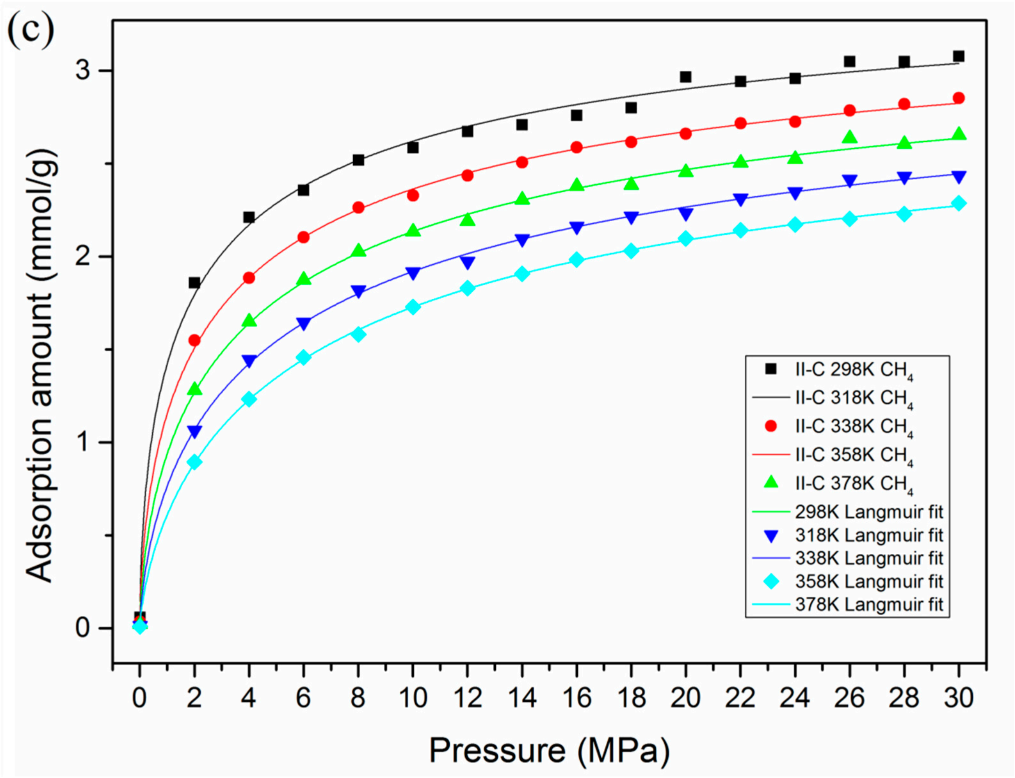
(c)
Adsorption amount (mmol/g)
Pressure (MPa)
0
1
2
3
0 2 4 6 8 10 12 14 16 18 20 22 24 26 28 30
II-C 298K CH4
II-C 318K CH4
II-C 338K CH4
II-C 358K CH4
II-C 378K CH4
298K Langmuir fit
318K Langmuir fit
338K Langmuir fit
358K Langmuir fit
378K Langmuir fit

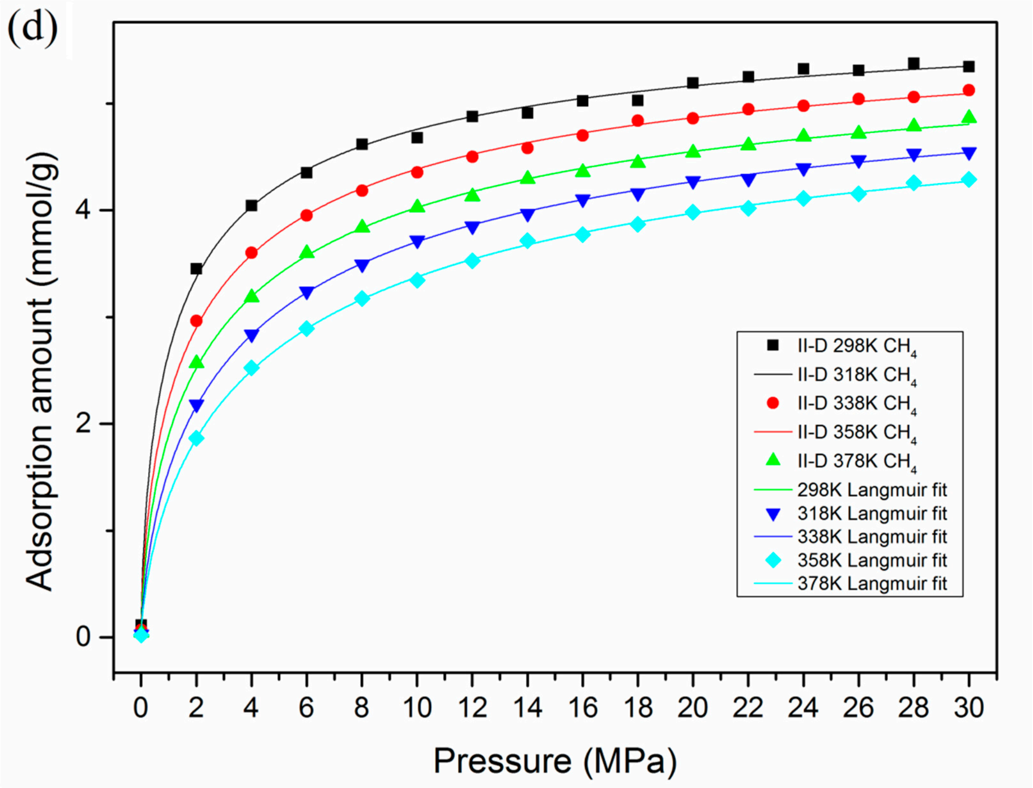

Figure S1. The adsorption isotherms of pure CH_4 at different temperatures and Langmuir fitting in kerogen with different maturity levels: (**a**) I-A; (**b**) II-B; (**c**) II-C; (**d**) II-D.

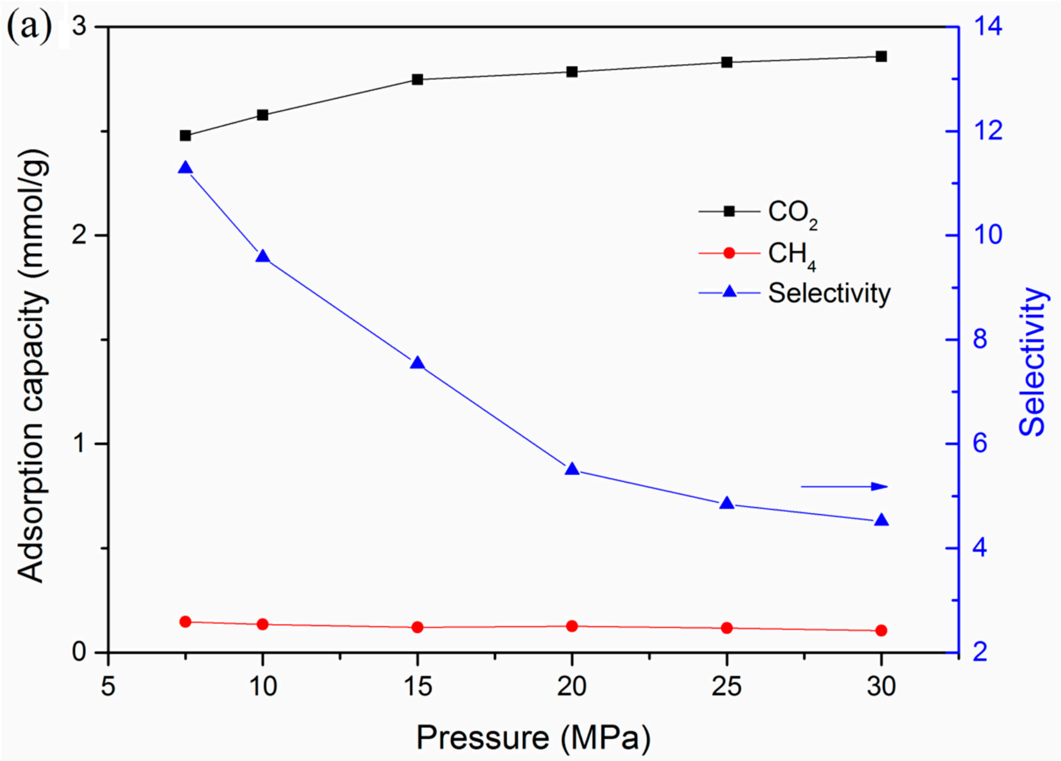

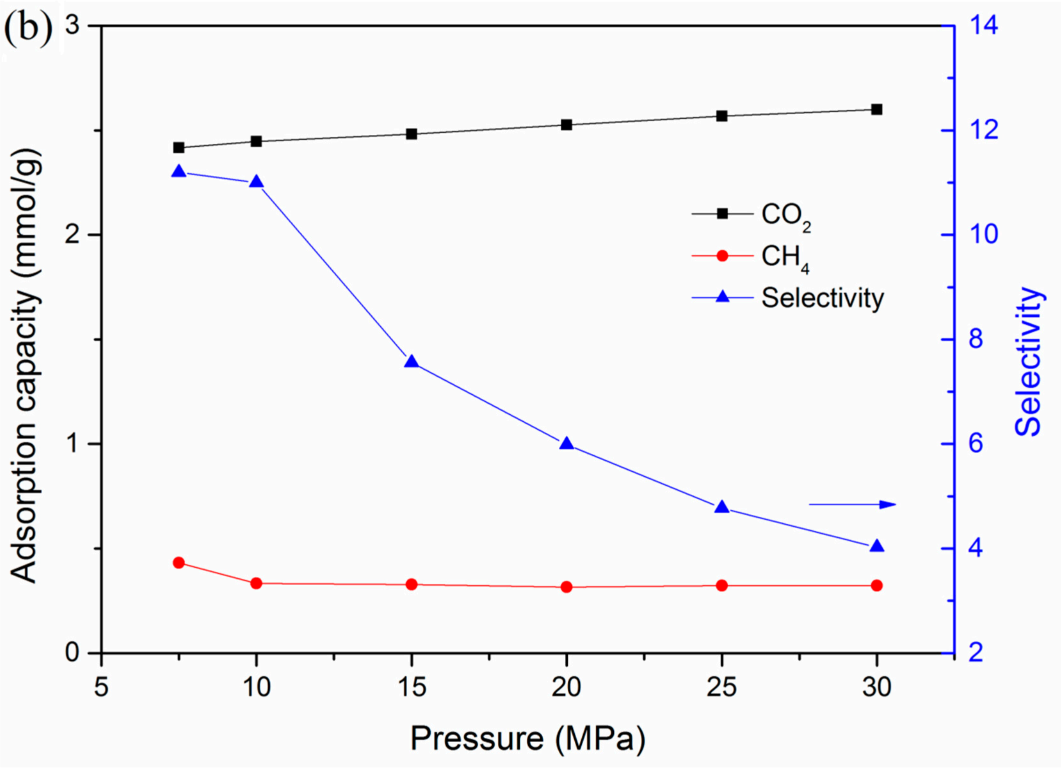

(b)
Adsorption capacity (mmol/g)
Selectivity
Pressure (MPa)
CO_2
CH_4
Selectivity
0
1
2
3
5
10
15
20
25
30
2
4
6
8
10
12
14

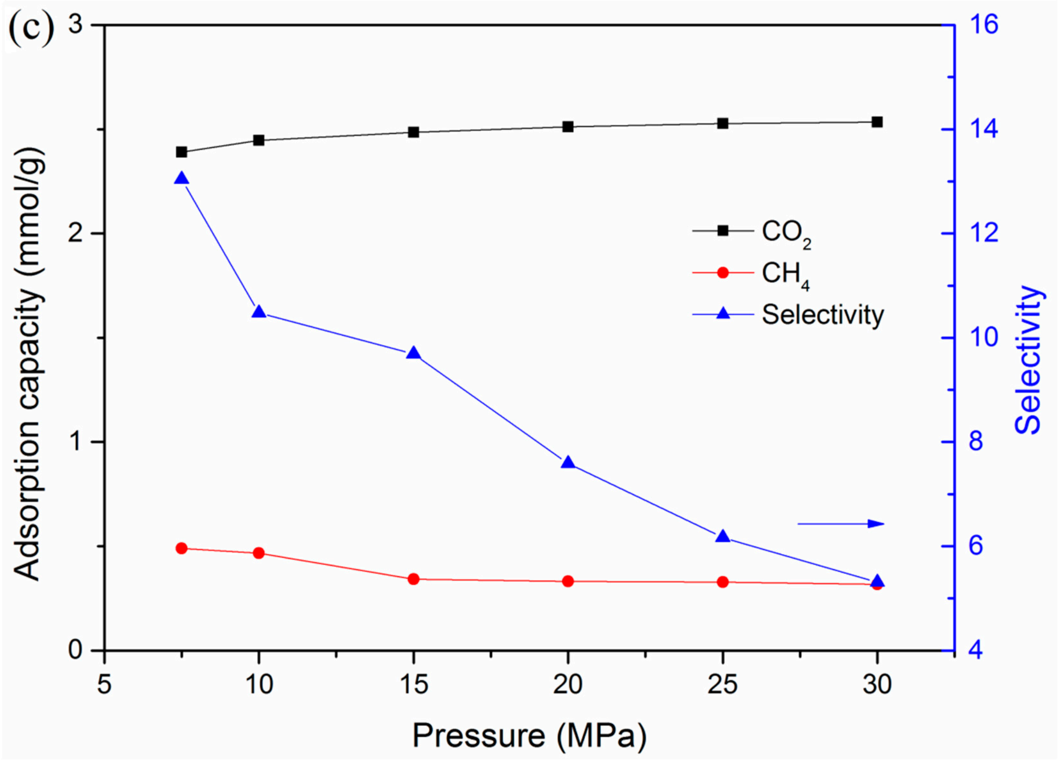

(c)
Adsorption capacity (mmol/g)
Selectivity
Pressure (MPa)
CO_2
CH_4
Selectivity
0
1
2
3
5
10
15
20
25
30
4
6
8
10
12
14
16

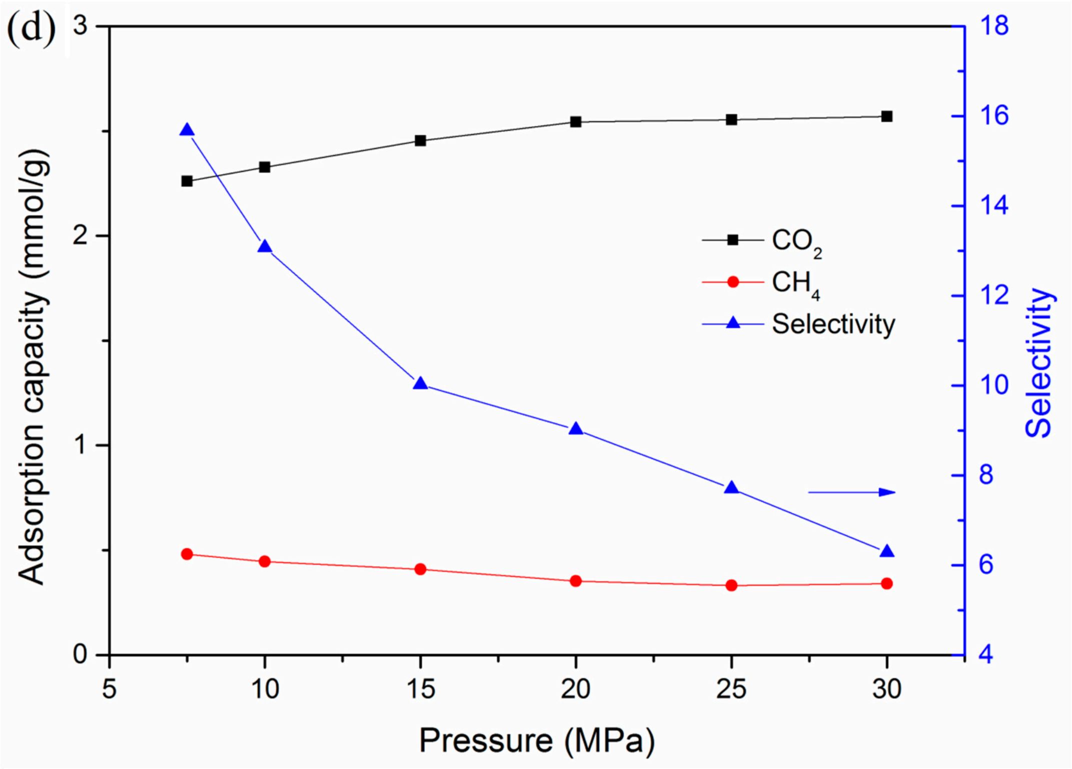

Figure S2. Variation of adsorption capacities and selectivity of CO_2/CH_4 binary mixture as a function of CO_2 partial pressure at different CH4 fixed partial pressures: (a) 5 MPa; (b) 15 MPa; (c) 20 MPa; (d) 25 MPa, T=298 K.
